# Case Report: Eosinophilic esophagitis and hen's egg tolerance—two cases, treated with topical steroids, showing remission with highly heated but not less heated hen's egg

**DOI:** 10.3389/falgy.2025.1475023

**Published:** 2025-03-06

**Authors:** Lisa Nuyttens, Liselot De Vlieger, Toon Ieven, Marianne Diels, Tessa Bosmans, Sara Van Meerbeeck, Tine Alliet, Toon Dominicus, Ilse Hoffman, Dominique M. A. Bullens

**Affiliations:** ^1^ Allergy and Clinical Immunology Research Group, Department of Microbiology, Immunology & Transplantation, KU Leuven, Leuven, Belgium; ^2^ Department of Pediatrics, University Hospitals Leuven, Leuven, Belgium; ^3^ Department of General Internal Medicine, Division of Allergy and Clinical Immunology, University Hospitals Leuven, Leuven, Belgium; ^4^ Pediatric Gastroenterology, Hepatology and Nutrition, University Hospitals Leuven, Leuven, Belgium

**Keywords:** PedEoE, hen’s egg-triggered EoE, heated hen’s egg, tolerance, case report

## Abstract

Eosinophilic esophagitis (EoE) is a chronic, antigen-mediated disease characterized by eosinophilic inflammation of the esophagus. Common triggers in Europe include cow's milk, hen's egg, wheat, soy, (pea)nuts, and (shell)fish. Recent studies indicate that heated forms of cow's milk in cow's milk-induced EoE may be well tolerated. Similar to cow's milk, we present two cases of children with hen's egg-triggered pediatric EoE treated with topical steroids who remained in remission after the introduction of highly heated forms of hen's egg. The introduction of less heated forms, however, led to relapse. These two cases suggest that heated hen's egg may be well tolerated in hen's egg-induced pediatric EoE, potentially allowing for a less restrictive diet and improved quality of life. Further research is necessary to explore the potential for inducing tolerance to less heated and/or raw egg through the gradual introduction of heated egg products.

## Introduction

Eosinophilic esophagitis (EoE) is a chronic, antigen-mediated disease characterized by localized eosinophilic inflammation of the esophagus. Symptoms vary depending on the child's age, with vomiting, nausea, and feeding difficulties resulting in failure to thrive being prominent in young children. Meal-associated cough, epigastric pain, dysphagia, and even food impaction are more prevalent in older children and adolescents (1, 2). The diagnostic criteria for EoE necessitate the presence of both clinical manifestations consistent with esophageal dysfunction and histological evidence of >15 eosinophils per high-power microscopy field (HPF) in esophageal biopsies (1). Treatment strategies include proton pump inhibitors (PPIs), topical corticosteroids (tCS), or elimination diets to achieve disease remission.

In Europe, the six most common food triggers are cow's milk (CM), hen's egg (HE), wheat, soy, (pea)nuts, and (shell)fish. Empirically, avoiding one or more of these allergens can often induce remission (3). However, recent findings suggest that in EoE triggered by cow's milk, baked milk products can in some cases be tolerated (4–6). Similar to cow's milk, we present two cases of hen's egg-triggered pediatric (Ped)EoE (while under treatment with tCS) that remained in remission after the introduction of highly heated forms of egg. Unfortunately, EoE relapse occurred after the introduction of less heated forms of hen's egg, which resulted in renewed exclusion of hen's egg from the children's diet.

## Case presentations

### Case 1

**Figure 1:**
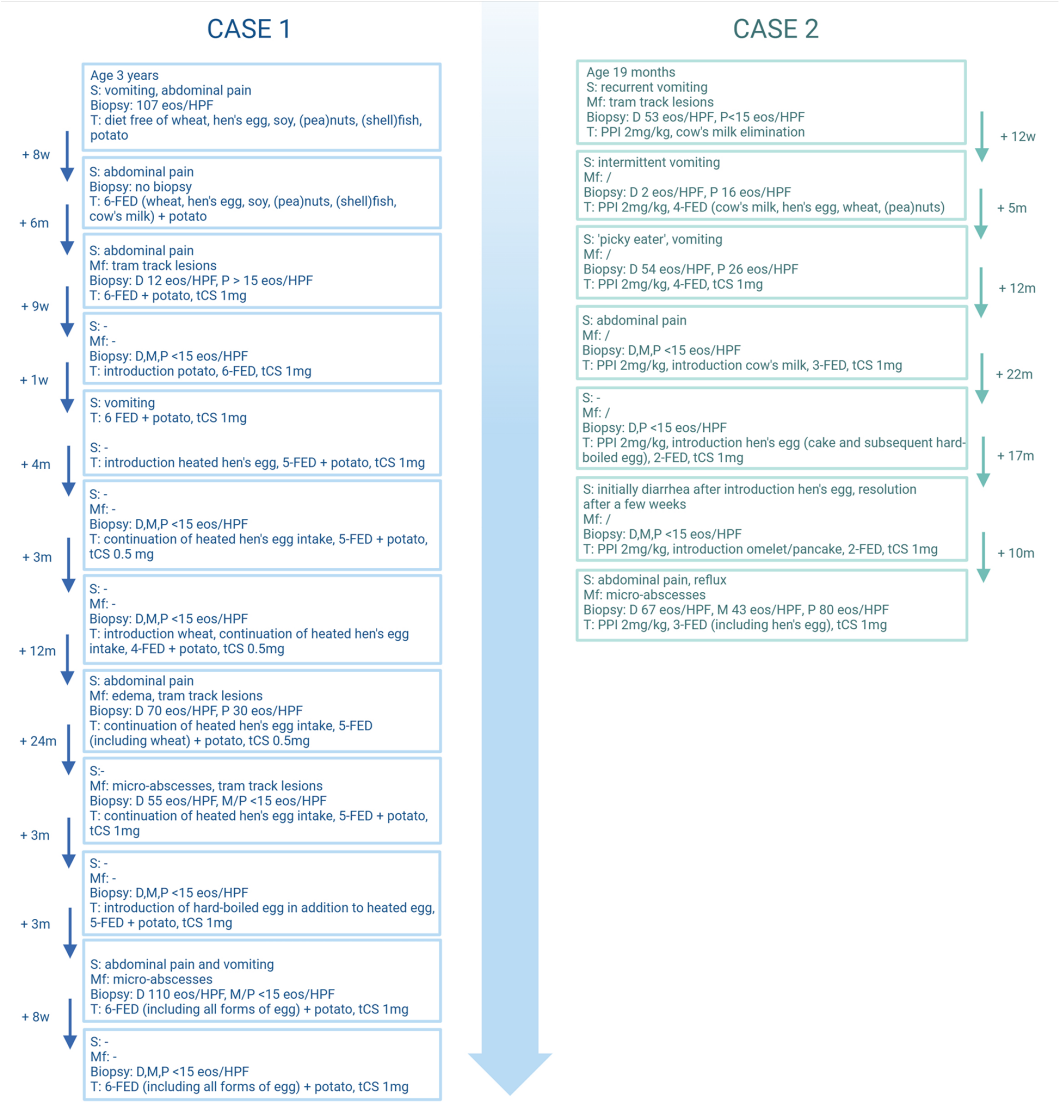
Schematic representation of the two cases. w, weeks; m, months; s, symptoms; Mf, macroscopic features; D, distal eosinophilic count (eos/HPF); M, mid eosinophilic count (eos/HPF); P, proximal eosinophilic count (eos/HPF); T, therapy; PPI, proton pump inhibitor; FED, food elimination diet; tCS, topical corticosteroid. Created in BioRender. (2025) https://BioRender.com/a24e976.

An 8-year-old boy was diagnosed with EoE at the age of 3 years due to vomiting and abdominal pain (Figure 1, Table 1). After the failure of a patient-tailored food elimination diet—including wheat, HE, soy, (pea)nuts, (shell)fish, CM, and potato [the latter based on an IgE-mediated allergy (7)]— tCS (viscous budesonide 1 mg/day, Box 1) were added to the treatment, resulting in remission. Eventually, heated HE was introduced in the form of a cake (heated 120°C, ≥30'; 1 slice 3×/week) in a rice flour-based form, adapted from the wheat-based cake recipe used in step 1 of our HE gradual introduction scheme (8) (Box 2). Persistent clinical and endoscopic remission was observed and tCS were successfully reduced to 0.5 mg/day. Subsequent wheat reintroduction to allow for the consumption of a wheat-based cake led to clinical and endoscopic relapse (70 eos/HPF). Since the re-elimination of wheat was insufficient to re-induce remission, tCS were increased to 1 mg/day with remission 12 weeks later. Hard-boiled HE (step 2 of the gradual protocol) (8) was then introduced after a successful in-hospital oral food challenge (ihOFC). After 1 week, the patient reported abdominal pain and vomiting with EoE recurrence confirmed by endoscopy (110 eos/HPF). An elimination diet excluding all forms of HE was reinstated, resulting in remission. The dose of tCS was maintained at 1 mg/day.

**Table 1:** Characteristics of the two cases.

Characteristic	Case 1	Case 2
(Specific) IgE (kU/L) at EoE diagnosis		
Total IgE	273	110
Hen's egg	0.67	0.36
Cow's milk	0.45	1.60
Soy	1.51	<0.10
Wheat	1.51	0.11
Peanut	1.31	NA
Tree nuts	NA	0.21
(Shell)fish	1.53	<0.10
Potato	1.4	NA
Diet and medications at first EoE remission		
Diet	Six-FED + potato	Four-FED [cow's milk, hen's egg, wheat, and (pea)nuts]
Medications	Budesonide 1 mg in Xanthan gum	PPI 2 mg/kg, budesonide 1 mg in Xanthan gum
Forms of heated egg tolerated		
Step 1: cake	Yes	Yes
Step 2: hard-boiled egg	No	Yes
Step 3: pancake, omelet	No	No
Step 4: soft-boiled egg	No	No
Step 5: raw egg	No	No

**Box 1 The dose and preparation of the tCS (budesonide in Xanthan gum)**

- Topical steroids are administered in a single dose.

The preparation of the two individual components is carried out by a pharmacist. Budesonide 0.5 mg/1 ml nebulizing suspension

Xanthan gum 600 mg, prepared as a suspension up to 100 ml

- The preparation is to be completed by the parents immediately prior to administration, following the instructions outlined below:

Prepare a solution by drawing 2 ml of budesonide (equivalent to 1 mg) into a syringe and adding Xanthan gum suspension to a final volume of 10 ml.

This preparation is to be administered each evening prior to bedtime. Following administration, no food or drink should be consumed.

**Box 2 Recipe cake (rice flour base)**

Ingredients

- 5 eggs
- 250 g rice flour
- 250 g sugar
- 250 g fat (butter)

Recipe

- Mix all ingredients together.
- Pour the dough into a greased baking tin (cake pan).
- Bake for at least 35 min in an oven at 160°C.

Notes

- The cake must be well-baked. Therefore, it is better to bake a thin, flat cake. Place the raw dough approximately 2–3 cm high in the baking pan and bake in several baking tins or a cake pan. If using an ordinary, tall, classic cake pan, use mainly the sides of the cake as the crust up to 2–3 cm wide is usually well-baked. It is best not to serve the inside as that may not be as well-baked.
- Do not incorporate fresh fruit in the cake (because around the fruit, the egg/dough will not be sufficiently well-baked).

### Case 2

A 7-year-old boy with recurrent vomiting was diagnosed with EoE at 19 months of age (Figure 1, Table 1). PPI therapy (2 mg/kg) was initiated, in part to also address concurrent reflux symptoms. After 3 months of PPI treatment combined with an IgE-directed elimination diet for CM, full endoscopic remission was not attained (16 eos/HPF) and a four-food elimination diet (FFED) including CM, HE, wheat, and (pea)nuts was undertaken, again without effect. Thus, 5 months later, after joint consultation with the parents, viscous budesonide 1 mg/day (Box 1) was added to the treatment, resulting in remission by the age of 3 years. Based on decreasing CM-specific IgE (1.60 to <0.10 kU/L), heated CM (20') was introduced, followed by progressively less heated CM and finally unheated CM after 14 months with persistent endoscopic remission. At the age of 5 years, heated HE was introduced (step 1, rice flour-based cake, Box 2) after a successful ihOFC. The cake was consumed several times per week without symptom recurrence. Furthermore, 6 months later, step 2 (hard-boiled HE) was introduced (8) and steadily increased to 1/2 portions/week (white and yolk) without symptoms. Endoscopy 8 weeks after hard-boiled HE introduction showed no recurrence. However, 4 weeks after step 3 (omelet and gluten-free pancake) (8), clinical and endoscopic recurrence was noted (80 eos/HPF), resulting in re-elimination of all forms of HE. The boy remained on PPIs and tCS (1 mg/day) throughout the gradual HE reintroduction. Afterward, an elimination diet excluding all forms of HE was reinstated; however, remission was not achieved. Remission was subsequently attained after increasing the tCS dose to 2 mg/day.

## Discussion

PedEoE is a heterogeneous disease that necessitates a tailored therapeutic approach. Historically, PPIs were considered the first-line treatment for EoE, with administration of tCS or dietary elimination reserved for cases in which remission was not achieved. However, the current guidelines now position PPIs, tCS, and dietary elimination as equally appropriate first-line therapeutic strategies (1). Histological remission is achieved in 59%–66% of children treated with tCS, in 30%–70% of children treated with PPIs, and in 42%–57.2% of children undergoing dietary interventions (1, 2). When dietary elimination is selected as the therapeutic approach, several strategies are available, each associated with different histological remission outcomes. Remission rates have been reported as ranging from 60.2% to 74% with a six-food elimination diet (SFED), from 56.3% to 64% with a FFED, from 30% to 67% with a one-food elimination diet (OFED), and from 52.6% to 58% with a targeted elimination diet (TED) (1, 9–11). In some cases, monotherapy may prove insufficient, necessitating a combination approach involving PPIs and/or dietary elimination and/or tCS to achieve optimal disease control (1). We have recently reviewed all PedEoE cases at our institution, highlighting the treatment difficulties in daily practice (12). Dietary elimination requires complete avoidance of the causative food. Adherence to such a diet is challenging due to the presence of allergenic protein in numerous (processed) foods. Furthermore, strict elimination diets may adversely impact both nutrition and overall quality of life (QoL), especially as more foods are restricted (8).

In the context of IgE-mediated food allergy, recent articles in the literature recommend heated allergen reintroduction, while avoiding less heated allergens during the tolerance introduction process. Indeed, emerging data show that a notable proportion (69%–83%) of children with an IgE-mediated CM allergy successfully tolerate baked CM products such as cake, whereas 63%–83% of children with IgE-mediated HE allergy tolerate baked HE products (4, 13). The allergenicity of CM and HE appears to be influenced by heating temperature and duration (8). Allowing the consumption of baked CM or HE products has the potential to improve compliance and enhance overall nutrition and QoL in children with a CM or HE allergy (4). Furthermore, we have provided evidence that consumption of heated HE may accelerate HE allergy resolution and that raw HE tolerance develops gradually through progressively less heated steps (e.g., cake, hard-boiled HE, omelet, and soft-boiled HE) (8).

Similar to what has been observed in IgE-mediated CM allergy, recent articles in the literature have shown that in a limited number of children with CM-triggered PedEoE, tolerance to baked CM was observed (4–6). In 2013, a retrospective analysis by Leung et al. showed that in a subgroup of children with CM-triggered EoE, 11/15 (73%) maintained histologic remission following the introduction of baked CM products (4). In 2019, Teoh et al. reported on a cohort of CM-triggered PedEoE with 24 children on a strict CM-free diet and 7 under a liberalized diet permitting the consumption of foods with traces of CM, including baked CM products. EoE remission was observed in 16/24 in the strict avoidance group and 2/7 in the liberalized group (5). Furthermore, González-Cervera et al. conducted a prospective study involving 18 patients with CM-triggered EoE in remission, where subjects consumed a minimum of 200 ml of sterilized CM (>100°C for 20') 2×/day over an 8-week period with 12/18 subjects maintaining endoscopic remission (6).

In contrast, Bianchi et al. reported on a 15-year-old who developed EoE during oral immunotherapy for a CM allergy and was initially permitted to eat baked CM products rather than adhering to a strict CM-free diet. Despite symptom resolution, a strict CM-free diet was ultimately required to induce histological remission (14).

Similar to the observed tolerance of heated CM in EoE, our two cases of HE-triggered PedEoE under treatment with tCS strongly suggest that heated HE may be well tolerated in HE-triggered PedEoE and that strict HE avoidance may not be universally required. In the first case, the introduction of a baked HE product (cake) resulted in the maintenance of EoE remission. Remarkably, the second case also tolerated a less heated form (hard-boiled HE) suggesting that in HE-triggered PedEoE, tolerance to less heated HE may be attainable.

The lower allergenic protein content of cake compared to a hard-boiled egg probably contributes to its reduced allergenicity. In the first case, it is plausible that reducing the amount of hard-boiled egg consumed may have been sufficient to maintain remission. However, the second case indicates that higher quantities of hard-boiled hen's egg may be tolerated in certain patients with HE-triggered PedEoE, while a less heated omelet in similar amounts was not tolerated.

Both patients received tCS therapy, which may have contributed to the maintenance of their remission. In case 1, the patient experienced a relapse following the reintroduction of wheat. tCS were reduced to 0.5 mg daily 3 months prior to this reintroduction. Unfortunately, remission did not occur after wheat was again eliminated but was only reestablished after the tCS dose was increased to 1 mg. This indicates that higher doses of tCS were needed to achieve remission. It is plausible that higher doses of tCS facilitated tolerance to the heated HE (rice flour cake), and that even this cake might not have been tolerated with a lower tCS dose. However, even at higher doses, less heated forms of hen's egg were not tolerated. In addition, it is clinically challenging to determine the exact timeframe during which therapeutic adjustments may still exert an effect (especially when multiple therapies are used concurrently). However, the literature suggests that an 8–12-week interval is generally considered sufficient to detect relapse on endoscopy (1). Complete withdrawal of tCS could, in this case, have indicated true tolerance to heated HE (cake), but this has not (yet) been performed.

In our two cases, tolerance acquisition may follow a gradual process analogous to that described in IgE-mediated HE allergy (8). This indicates that, on an individual basis, searching for the extent of HE tolerance in children with HE-triggered EoE may be worthwhile, and that further research is required to assess the extent to which complete raw HE tolerance can be achieved in children with HE-triggered EoE.

## Conclusion

We presented two cases of successful heated HE reintroduction in children diagnosed with HE-triggered PedEoE under concomitant treatment with a tCS, while less heated forms were not tolerated, drawing parallels with the observed tolerance of baked CM products in children with CM-triggered EoE. Further research is imperative to investigate the potential for raw HE tolerance induction by introducing these HE products and to understand whether this tolerance acquisition follows a gradual process.

## Data Availability

The original contributions presented in the study are included in the article/Supplementary Material, further inquiries can be directed to the corresponding author.
